# Conditional stimulus choices affect fear learning: Comparing fear conditioning with neutral faces and shapes or angry faces

**DOI:** 10.1111/psyp.14068

**Published:** 2022-04-27

**Authors:** Luke J. Ney, Camilla C. Luck, Allison M. Waters, Ottmar V. Lipp

**Affiliations:** ^1^ School of Psychology and Counselling Queensland University of Technology Brisbane Queensland Australia; ^2^ School of Population Health Curtin University Perth Western Australia Australia; ^3^ School of Applied Psychology Griffith University Brisbane Queensland Australia

**Keywords:** conditional stimulus, CS pleasantness, electrodermal responses, fear conditioning

## Abstract

Past fear conditioning studies have used different types of conditional stimuli (CSs). Whether this choice affects learning outcomes in particular when neutral stimuli (e.g., neutral faces vs. shapes) are used is unclear. Data were aggregated across nine studies using an electric shock unconditional stimulus to test for differences in acquisition and extinction of electrodermal responses and self‐reported CS pleasantness when CSs were neutral faces or shapes (Experiment 1, *N* = 594) and when CSs were angry or neutral faces (Experiment 2, *N* = 157). Reliable electrodermal conditioning was observed in all stimulus conditions. We found stronger differential conditioning in electrodermal second interval responses and CS pleasantness and more pronounced extinction in CS pleasantness for neutral shape than neutral face CSs, but no differences in electrodermal first interval responses, the most frequently reported index of fear conditioning. For angry and neutral face CSs, there were no differences during acquisition, but the extinction of first and second interval electrodermal conditioning to angry faces was retarded relative to neutral faces. Acquisition of differential CS pleasantness, which was reliably observed for neutral face CSs, was absent for angry face CSs. The current results suggest that fear conditioning with a neutral face and shape CSs yields broadly similar results with differences limited to second interval electrodermal responses and CS pleasantness ratings. Using angry face CSs resulted in impaired extinction of electrodermal indices and no differential CS pleasantness ratings and should only be considered in studies designed to address questions about these specific CS materials.

## Abstract 2

Methodological choices like the nature of the conditional stimulus (CS) may affect fear learning. Here, we document in a very well powered analysis, that differences in fear conditioned to neutral face or shape CSs are limited to anticipatory electrodermal responses and self‐reported CS pleasantness but are not apparent in the more frequently reported electrodermal responses to CS onset. In contrast, differences in fear conditioned to angry and neutral face CSs emerge across indices of fear, in particular during extinction.

## 1 INTRODUCTION

Experimental psychology has long established that when a neutral stimulus is repeatedly paired with a biologically relevant outcome, it becomes a conditional stimulus (CS) that can elicit a response even in absence of this outcome. The association between a CS and a feared outcome is fundamental to anxiety and trauma‐related disorders (Mineka & Zinbarg, 2006). Classical conditioning, first described by Pavlov (1927), posits that repeated pairing of a CS with an aversive stimulus results in a conditional fear response to the CS alone, which subsequently can be reduced through fear extinction learning (Bouton, 2004; Craske et al., 2014). Since this model of fear emphasizes associative learning, an early assumption was that the nature of the CS was irrelevant to the strength of the association that developed following acquisition—the equipotentiality principle (Öhman et al., 1976). However, this view was challenged by the concepts of belongingness (Garcia & Koelling, 1966) and “preparedness” (Seligman, 1971), which posit that the pairings of some stimuli should result in stronger learning than that of others. Seligman (1971) argued that certain associations are biologically prepared to result in faster acquisition, slower extinction, and immunity from cognitive instruction.

Previous research has provided varying support for the preparedness theory of fear and extinction learning. Some studies have found that animal fear‐relevant stimuli (e.g., spiders and snakes) display more rapid fear acquisition (Ho & Lipp, 2014; Öhman et al., 1975) and impaired extinction learning (Öhman et al., 1975, 1976) compared to non‐fear relevant stimuli, however, these effects are not universally replicated (Åhs et al., 2018; de Jong & Merckelbach, 1997; Merckelbach et al., 1987). Preparedness effects have similarly been reported for face stimuli, with angry faces producing stronger acquisition learning and impaired extinction learning compared to neutral faces (Dimberg & Öhman, 1996; Esteves et al., 1994; Hamm et al., 1989; Öhman & Dimberg, 1978; Rowles et al., 2012), but only if pictures of adult males are used (Mazurski et al., 1996; Öhman & Dimberg, 1978) with the poser facing toward the viewer (Dimberg & Öhman, 1983).

However, more recent evidence suggests that fear conditioned to fear‐relevant face or animal stimuli is not resistant to instructed fear extinction (Luck et al., 2020; Mallan et al., 2013; Pitman & Orr, 1986), which contradicts Seligman's (1971) notion of a qualitatively different “prepared” learning. Moreover, a recent systematic review found that only one‐third of published studies found support for increased resistance to extinction of fear conditioned to animal fear‐relevant CSs, which strongly challenges the preparedness theory, at least in the context of experimental fear conditioning and extinction (Åhs et al., 2018). Nevertheless, differences in the speed of acquisition and extinction of Pavlovian conditioning as a function of CS salience are well documented (Kamin, 1965) supporting the notion of a quantitative difference in learning as a function of CS materials.

Increasing recognition of the importance of producing replicable studies has resulted in attention to methodological details within fear conditioning experiments (Lonsdorf et al., 2017; Ney et al., 2020; Ryan et al., 2021). It is generally advised that biologically fear‐relevant CSs should be used only in cases where they are intended for experimental manipulation (Lonsdorf et al., 2017). It also has been suggested that “neutral” faces may not be neutral to all participants but are processed like unpleasant stimuli by participants, for instance, high in self‐reported social anxiety (Lange et al., 2012). However, there are no clear recommendations for the selection of different types of conditional stimuli in fear conditioning research due, in part, to a lack of empirical evidence. Although there is some research concerning whether fear conditioning with angry faces differs from that with neutral faces (Mallan et al., 2013) there is no published research to our knowledge examining whether fear conditioning with neutral faces differs from that with biologically fear‐irrelevant stimuli such as shapes, which are also commonly used CSs in fear conditioning (Ryan et al., 2019). Therefore, decision‐making in experimental design does not currently have an empirical basis when choosing between these types of stimuli as CSs.

The current study is a secondary analysis of data from several studies conducted previously by our laboratory group. In these studies, the CSs used were angry and neutral faces, as well as neutral shapes. In Experiment 1, we compare the acquisition and extinction of differential electrodermal responses and self‐reported CS pleasantness between neutral face and shape CSs. In Experiment 2, we compare the acquisition and extinction of differential electrodermal responses and self‐reported CS pleasantness between angry faces and neutral faces. We conducted these analyses so that future studies can make evidence‐based decisions when selecting CSs in fear conditioning and extinction studies. Given that neutral faces are not regarded as biologically fear‐relevant stimuli (Mallan et al., 2013), we hypothesized that neutral face and shape CSs would produce equivalent acquisition and extinction across all measures of conditioning, the primary measure of electrodermal first interval electrodermal responses as well as in second interval electrodermal responses and CS pleasantness ratings. Differential conditioning to angry faces would be acquired faster and be more resistant to extinction compared to neutral faces across all measures.

## 2 EXPERIMENT 1

### 2.1 Method

#### 2.1.1 Data and participants

Data from seven studies (Luck & Lipp, 2016; six currently unpublished [HREC approval numbers HR23/2014; HRE2017‐0537‐09; HRE2019‐0044]) using either shapes or neutral faces as CSs were compiled for Experiment 1. These studies had identical habituation and acquisition phases and five studies had identical extinction phases which commenced immediately after acquisition. One of the remaining studies provided instructions prior to extinction training (Luck & Lipp, 2016) and the second employed an evaluative counter‐conditioning procedure prior to extinction, hence, their extinction data were not included. Thus, 594 participants (300 trained with shapes; 286 males and 407 females, aged 17–72; mean = 22.68, *SD* = 6.26; one participant each did not provide age or gender information), provided electrodermal data from habituation and acquisition and 402 participants (252 trained with shapes; 114 males and 287 females, aged 17–52; mean = 21.94, *SD* = 5.26; one participant each did not provide age or gender information) provided electrodermal data from extinction. CS pleasantness evaluations were not available for three participants from habituation and acquisition and six participants from extinction. Participants were recruited from University cohorts, participated in exchange for course credits or a cash reimbursement, and provided informed consent.

#### 2.1.2 Apparatus and materials

Face CSs were four pictures of adult male Caucasians with neutral expressions taken from the NimStim database (Tottenham et al., 2009). The posers used differed across the studies and each participant saw two faces, counterbalanced across participants. Four geometric shapes (circle, square, triangle, and diamond; black outlines on a white background) were used as the shape CSs, with each participant seeing two shapes, counterbalanced across participants. All CSs were presented for 6 s on a 17‐inch computer monitor. Physiological data (skin conductance and respiration) were recorded using a Biopac MP150 system (sampled at 1000 Hz) and Acknowledge v4.1 software. Skin conductance was amplified at a gain of 5μSiemens/volt and respiration was recorded as a control measure. CS evaluations were collected continuously using a TSD115 variable assessment transducer with a 10‐point Likert scale (anchors: 0 = “very unpleasant”; midpoint = “neutral”; 9 = “very pleasant”). A 200 ms electro‐tactile stimulus that was pulsed at 50 Hz through a Grass SD9 stimulator or a sequence of three 2 ms electro‐tactile stimuli generated by a Digitimer DS7A stimulator unit presented 16 ms apart (perceived as one discrete stimulus) were used as the experimental US. The experimental stimulus sequence was controlled by DMDX software (Forster & Forster, 2003).

#### 2.1.3 Procedure

Upon arrival at the laboratory, participants were instructed to wash their hands and were seated in front of the computer monitor in a quiet room. They provided informed consent before having two 8‐mm Ag/AgCl pre‐gelled electrodes (Biopac EL507) attached to the thenar and hypothenar eminences of their non‐dominant hand, a respiration belt fitted around their waist, and a shock electrode attached to their dominant forearm. Next, a shock calibration procedure was performed to determine a US intensity that was subjectively “unpleasant, but not painful.” In brief, starting from zero, stimulus intensity was increased in steps of 5 V (SD9)/10 mA (DS7A) until the participants reported noticing the stimulus. These increments were continued until participants reported the stimulus intensity as unpleasant, but not painful. The final stimulation intensity was repeated to confirm the assessment and used throughout the experiment. Three minutes of baseline physiological data were then collected. Participants were then instructed to use the variable assessment transducer to indicate their evaluation of the CS displayed on the computer screen by moving the slider to a position that best represented their evaluation and then to move the slider back to the midpoint of the scale. These data were collected as a deviation of the electrical signal from the midpoint (zero Volts) of the scale. The experimenter confirmed that participants understood the instructions before commencing the stimulus sequence.

At the beginning of the experiment, participants completed a habituation phase consisting of four presentations of the CS+ and four presentations of the CS−, with the allocation of CS pictures to each category counterbalanced across participants. Participants were not instructed as to the CS–US contingency or the nature of each experimental phase at any point. Acquisition commenced immediately after habituation and comprised eight presentations of the CS+ and eight presentations of the CS− with the CS+ followed by the electric shock at 100% reinforcement, which co‐terminated with the CS+. Following the acquisition, participants in five of the seven studies proceeded immediately to an extinction phase, where the CS+ and CS− were presented eight times each without any electrical stimulation. Skin conductance and CS evaluations were collected throughout the experiment. Presentation of CS+/CS− was organized in a pseudo‐random fashion throughout the experiment, with neither the CS+ nor CS− presented more than twice consecutively. Intertrial interval duration varied between 16 and 20 s (CS offset to CS onset). A post‐experimental questionnaire was used to assess CS–US contingency awareness and ratings of CS and US pleasantness.

Data from 594 participants were included in the habituation and acquisition analysis (300 for Shapes, 294 for faces), and data from 402 participants (252 for Shapes and 150 for faces) were included in the extinction analysis. The number of included participants was not equal between acquisition and extinction because two of the seven studies involved specific experimental manipulations during extinction.

#### 2.1.4 Data scoring and analysis

Skin conductance responses were scored as the largest response starting within one of three latency windows following CS onset and the response magnitude was calculated as the difference between response onset and peak. Scores were then square root transformed and range corrected using the largest response observed for a particular participant across the entire experiment, usually the response to the first or second US, as reference (Dawson et al., 2016). Skin conductance responding was scored in three latency windows: First interval responding (FIR) was scored as the largest response starting within 1–4 s following CS onset, second interval responding (SIR) was scored as the largest response starting within 4–7 s following CS onset, and third interval responding (TIR) was scored as the largest response starting within 7–11 s following CS onset (Luck & Lipp, 2016; Prokasy & Ebel, 1967). Only FIRs were scored during habituation because FIRs reflect orienting to novel stimuli (Öhman, 1974). Online evaluations of CS pleasantness (provided by participants using the sliding transducer) were scored as the largest deviation in Volts from a zero Volt baseline during the presentation of a CS (Range − 2.5 V–2.5 V). Positive and negative values correspond to assessments of unpleasant and pleasant, respectively.

Prior to analysis, skin conductance responses and CS evaluations were averaged to produce block scores of two consecutive CS+ or CS− trials. Data were analyzed using repeated measures ANOVAs in SPSS v27, with partial eta squared (${\eta_{p}}^{2}$) reported as a measure of effect size. Each phase and latency window were analyzed separately with CS‐type (faces vs. shapes) × CS (CS+, CS−) × block mixed factorial ANOVAs with CS‐type as a between‐subject factor and repeated measures on the last two factors. The results of the multivariate solution (Phillai's trace) are reported as it does not necessitate the assessment of sphericity (Vasey & Thayer, 1987), and the significance level was set to .05. For each analysis, the highest order interaction effect was followed up with further analyses, *t* tests, or ANOVAs as required, without further adjustment of the overall level of significance.

## 3 RESULTS

### 3.1 Preliminary analyses

The neutral face group (198 females/95 males) did not differ from the shape group (209 females/91 males) in terms of gender distribution, *χ* ^2^ = 0.30, *p* = .583, rated US pleasantness (face group: *M* = −1.03, *SD* = 1.80; shape group: *M* = −1.09, *SD* = 1.74) or number of spontaneous skin conductance responses during baseline (face group: *M* = 19.28, *SD* = 14.09; Shape group: *M* = 20.24, *SD* = 14.09), both *t* < 0.82, *p* > .412. However, the group trained with Shape CSs had a higher number of participants who (a) failed to report the CS–US contingencies (44 out of 300 vs. 21 out of 294), *χ* ^2^ = 8.63, *p* = .003, (b) were younger (Shape group: *M* = 22.11, *SD* = 6.46; face group: *M* = 23.27, *SD* = 6.00), *t*(591) = 2.27, *p* = .024, and (c) displayed larger maximal skin conductance responses, the response used as reference for range corrections (Shape group: *M* = 1.95 μS, *SD* = 0.75; face group: *M* = 1.81 μS, *SD* = 0.65), *t*(592) = 2.47, *p* = .014. We assessed whether these group differences affected the outcomes of the current study by running preliminary analyses that included these variables as covariates and report if the inclusion of a covariate affected effects involving the between groups factor.

### 3.2 Electrodermal first interval responses

**FIGURE 1:**
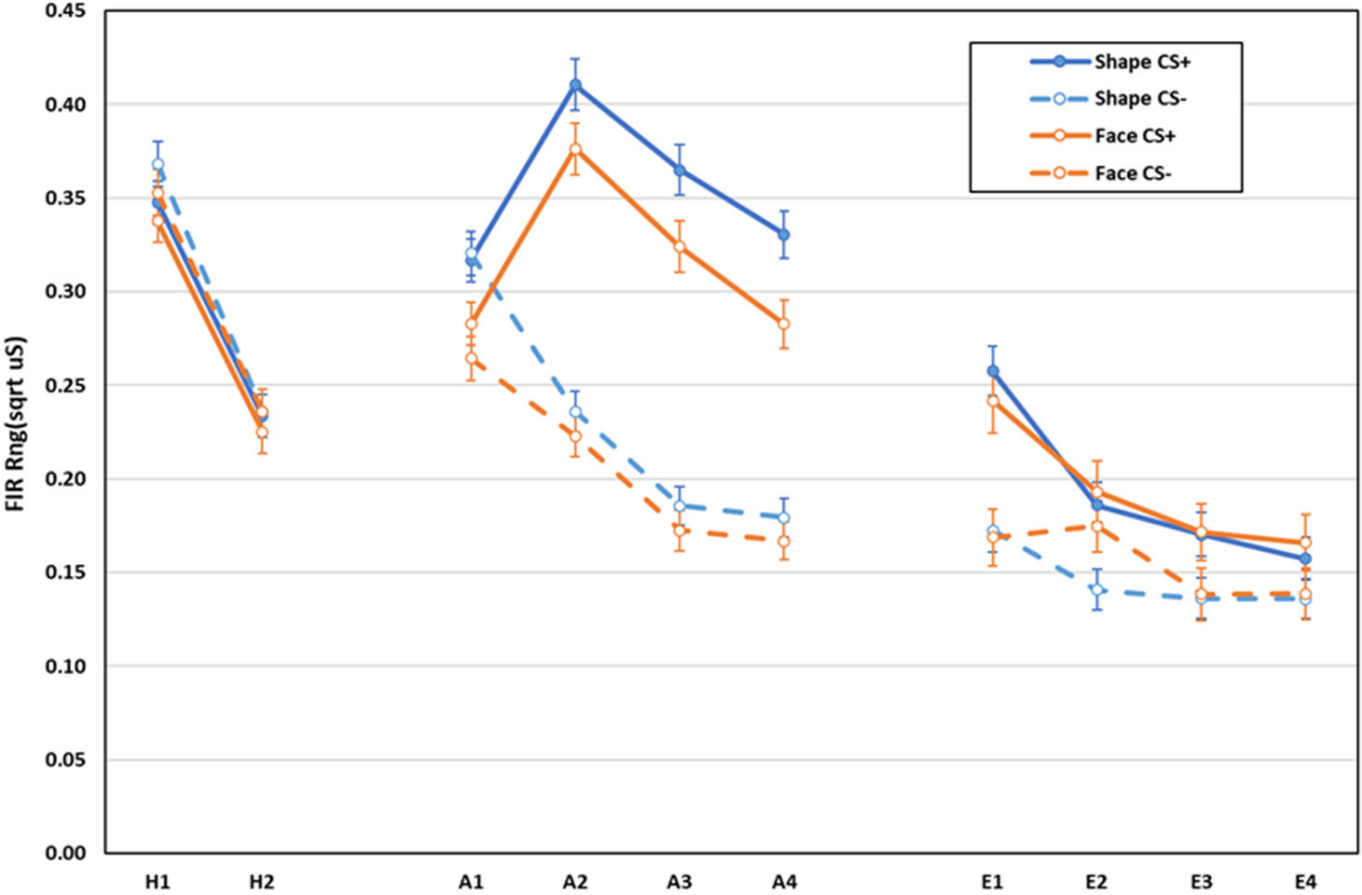
Electrodermal first interval responses during habituation, acquisition, and extinction training as a function of CS‐type (shapes vs. neutral faces), CS (CS+ vs. CS−), and blocks of 2 trials (error bars represent standard errors of the mean)

Electrodermal first interval responses during habituation, acquisition, and extinction are displayed in Figure 1. During habituation, responses to CS− were larger than to CS+, CS main effect, *F*(1,592) = 4.13, *p* = .043, ${\eta_{p}}^{2}$ = .007, and responding reduced between blocks, main effect block, *F*(1,592) = 417.81, *p* < .001, ${\eta_{p}}^{2}$ = .414. All other effects were non‐significant, *F* < 1.00, *p* > .332, ${\eta_{p}}^{2}$ < .003.

During acquisition, Shapes elicited larger responses overall than faces, main effect CS‐type, *F*(1,592) = 6.62, *p* = .010, ${\eta_{p}}^{2}$ = .011. Main effects for CS, *F*(1,592) = 416.06, *p* < .001, ${\eta_{p}}^{2}$ = .413, and block, *F*(3,590) = 48.37, *p* < .001, ${\eta_{p}}^{2}$ = .197, were qualified by a CS × block interaction, *F*(3,590) = 72.24, *p* < .001, ${\eta_{p}}^{2}$ = .269, with no difference in responding to CS+ and CS− on block 1, *F*(1,592) = 0.79, *p* = .375, ${\eta_{p}}^{2}$ = .001, but larger responses to CS+ than to CS− on blocks 2–4, all *F*(1,592) > 224.17, *p* < .001, ${\eta_{p}}^{2}$ > .274. All other effects were non‐significant, *Fs* < 2.03, *p* > .108, ${\eta_{p}}^{2}$ < .011.

During extinction, main effects for CS, *F*(1,400) = 75.27, *p* < .001, ${\eta_{p}}^{2}$ = .158, and block, *F*(3,398) = 21.18, *p* < .001, ${\eta_{p}}^{2}$ = .138, were qualified by a CS × block interaction, *F*(3,398) = 8.66, *p* < .001, ${\eta_{p}}^{2}$ = .061. Responses to CS+ were larger than to CS− on blocks 1–4, all *F*(1,400) > 7.82, *p* < .006, ${\eta_{p}}^{2}$ > .018, with the difference significantly larger on block 1 (*M* = 0.081, *SD* = 0.17) than on the others (all *M* < 0.036, *SD* <0.18, all *p* < .001). All other effects were non‐significant: all *F* < 165, *p* > .178, ${\eta_{p}}^{2}$ < .013.

### 3.3 Electrodermal second interval responses

**FIGURE 2:**
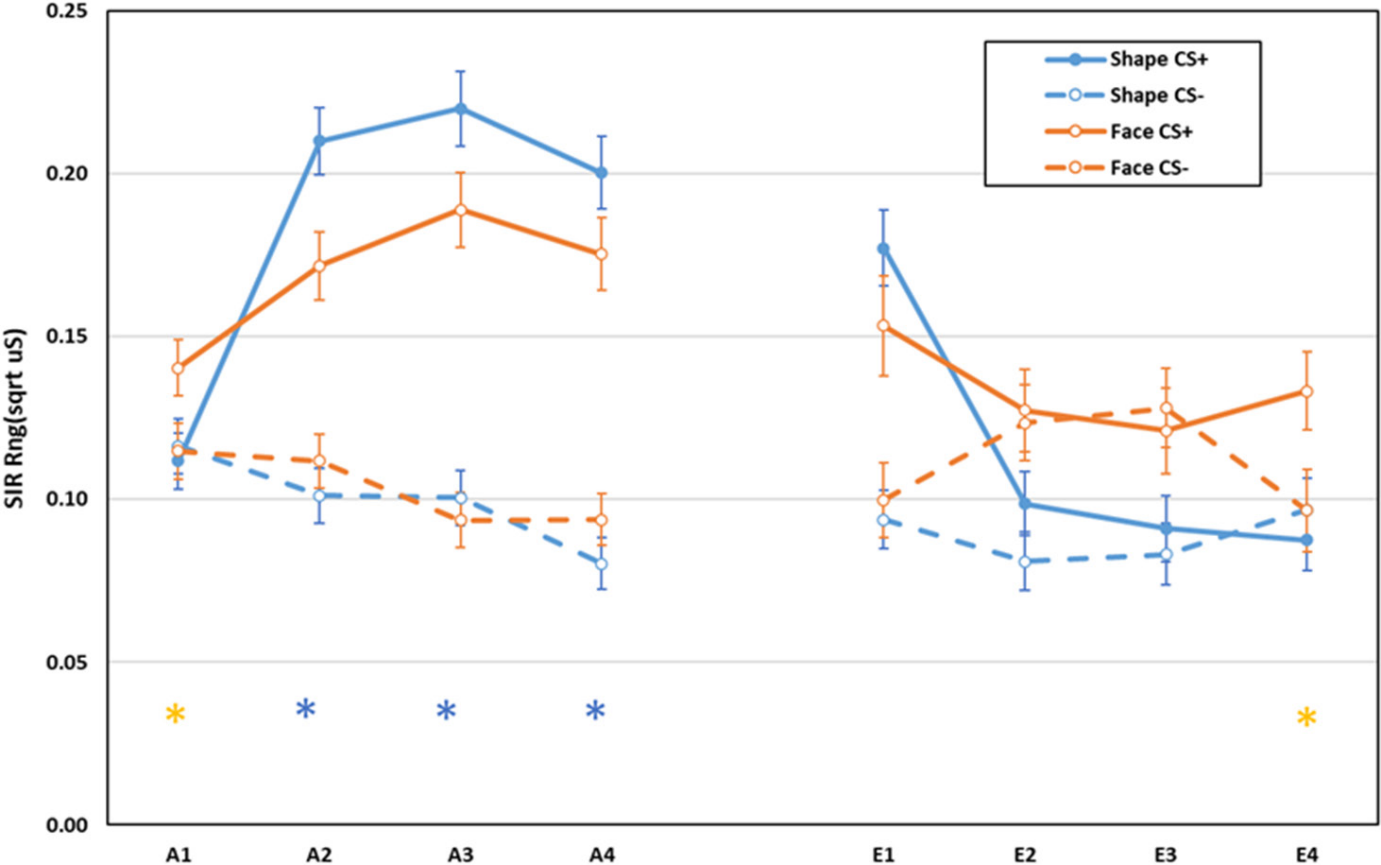
Electrodermal second interval responses during acquisition and extinction training as a function of CS‐type (shapes vs. neutral faces), CS (CS+ vs. CS−), and blocks of 2 trials (error bars represent standard errors of the mean; * indicate larger differential responding in group faces 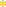 or shapes 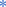 )

Electrodermal second interval responses during acquisition and extinction are displayed in Figure 2. During acquisition, main effects for CS, *F*(1,592) = 238.02, *p* < .001, ${\eta_{p}}^{2}$ = .287, and block, *F*(3,590) = 12.35, *p* < .001, ${\eta_{p}}^{2}$ = .059, were qualified by a CS × block, *F*(3,590) = 28.64, *p* < .001, ${\eta_{p}}^{2}$ = .127, CS‐type × CS, *F*(1,592) = 4.29, *p* = .039, ${\eta_{p}}^{2}$ = .007,^1^ CS‐type × block, *F*(3,590) = 3.23, *p* = .022, ${\eta_{p}}^{2}$ = .016, and CS‐type × CS × block interactions, *F*(3,590) = 4.87, *p* = .002, ${\eta_{p}}^{2}$ = .024. Breakdown of the three‐way interaction revealed that for participants trained with Shapes there was no significant difference in responses to CS+ and CS− on block 1, *F*(1,592) = 0.19, *p* = .663, ${\eta_{p}}^{2}$ < .001, but the CS+ elicited larger responses than CS− on blocks 2–4, all *F*(1,592) > 87.20, *p* < .001, ${\eta_{p}}^{2}$ > .127. For participants trained with faces, CS+ elicited larger responses than CS− across all blocks, all *F*(1,592) > 5.97, *p* < .016, ${\eta_{p}}^{2}$ > .009. Differential responding was larger for the face than Shape CSs on block 1 (face group: *M* = 0.03, *SD* = 0.18; Shape group: *M* = −0.005, *SD* = 0.18, *p* = .041), but larger for the Shape CSs on blocks 2 and 4 (block 2: face group: *M* = 0.06, *SD* = 0.20; Shape group: *M* = 0.11, *SD* = 0.21, *p* = .003; block 3: face group: *M* = 0.10, *SD* = 0.21; Shape group: *M* = 0.12, *SD* = 0.21, *p* = .166; block 4: face group: *M* = 0.08, *SD* = 0.21; Shape group: *M* = 0.12, *SD* = 0.22, *p* = .030). All other effects were non‐significant, *F* < 1.0, *p* > .437, ${\eta_{p}}^{2}$ < .002.

During extinction, main effects for CS‐type, *F*(1,400) = 4.41, *p* = .036, ${\eta_{p}}^{2}$ = .011,^2^ CS, *F*(1,400) = 17.37, *p* < .001, ${\eta_{p}}^{2}$ = .042, and block, *F*(3,398) = 6.37, *p* < .001, ${\eta_{p}}^{2}$ = .046, were qualified by CS‐type × block, *F*(3,398) = 5.22, *p* = .002, ${\eta_{p}}^{2}$ = .038, CS × block: *F*(3, 398) = 11.78, *p* < .001, ${\eta_{p}}^{2}$ = .082, and CS‐type × CS × block interactions, *F*(3, 398) = 3.97, *p* = .008, ${\eta_{p}}^{2}$ = .029. Shape CSs+ elicited larger responses than shape CSs− on block 1, *F*(1,400) = 48.04, *p* < .001, ${\eta_{p}}^{2}$ = .107, but there was no differential responding on blocks 2–4, all *F*(1,400) < 2.59, *p* > .107, ${\eta_{p}}^{2}$ < .007. For face CSs, there was differential responding on blocks 1 and 4, both *F*(1,400) > 7.09, *p* < .009, ${\eta_{p}}^{2}$ > .016, but not on blocks 2 or 3, both *F*(1,400) < 1.0, *p* > .644, ${\eta_{p}}^{2}$ < .002. All other effects were non‐significant: *F* < 1, *p* > .775, ${\eta_{p}}^{2}$ < .001. The extent of differential responding did not differ between groups on blocks 1–3, all *F*(1,400) < 2.33, *p* > .127, ${\eta_{p}}^{2}$ < .007, but was larger in group Faces than in group Shapes on block 4, *F*(1,400) = 6.99, *p* < .009, ${\eta_{p}}^{2}$ = .017.

### 3.4 Electrodermal third interval responses

**FIGURE 3:**
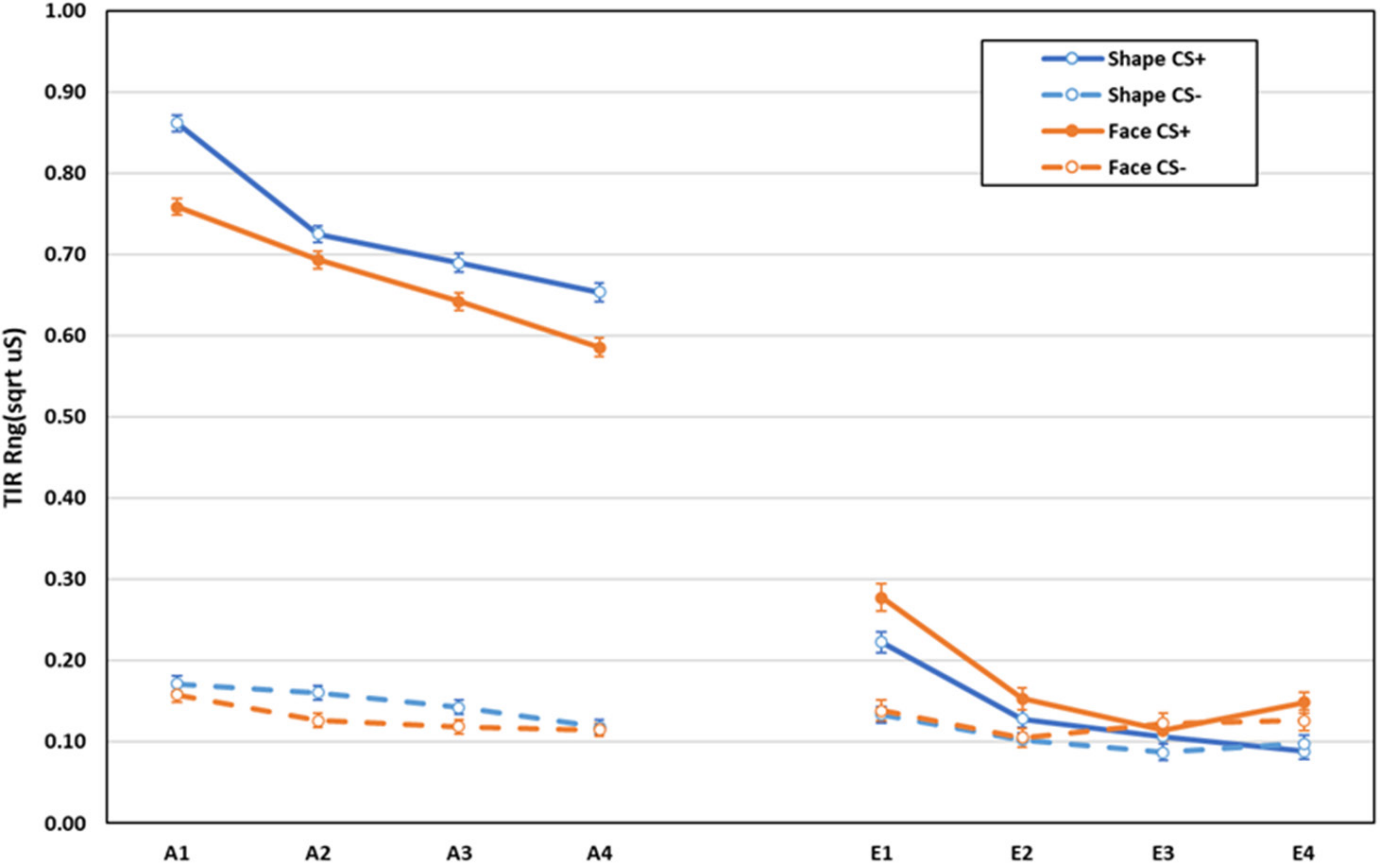
Electrodermal third interval responses during acquisition and extinction training as a function of CS‐type (shapes vs. neutral faces), CS (CS+ vs. CS−), and blocks of 2 trials (error bars represent standard errors of the mean)

Electrodermal third interval responses during acquisition and extinction are displayed in Figure 3. During acquisition, main effects for CS‐type, *F*(1,589) = 26.62, *p* < .001, ${\eta_{p}}^{2}$ = .043, block, *F*(3,587) = 135.16, *p* < .001, ${\eta_{p}}^{2}$ = .409, and CS, *F*(1,589) = 6303.17, *p* < .001, ${\eta_{p}}^{2}$ = .915, were qualified by CS‐type × CS, *F*(1,589) = 9.55, *p* = .002, ${\eta_{p}}^{2}$ = .016, CS × block, *F*(3,587) = 45.15, *p* < .001, ${\eta_{p}}^{2}$ = .187, and CS‐type × CS × block interactions, *F*(3,587) = 7.17, *p* < .001, ${\eta_{p}}^{2}$ = .035. Breakdown of the three‐way interaction revealed that responses to the US after the CS+ significantly reduced across blocks for both shapes and faces (block 1 > block 2 > block 3 > block 4, all *p* < .001), whereas responses after CS− reduced at different rates (face group: block 1 > block 2, block 3, block4; shape group: block 1 > block 3, block4; block 2 > block 4; all *p* < .01). All other effects were non‐significant, *F* < 1.98, *p* > .116, ${\eta_{p}}^{2}$ < .011.

During extinction, main effects for CS‐type, *F*(1,400) = 6.23, *p* = .013, ${\eta_{p}}^{2}$ = .015, CS, *F*(1,400) = 52.53, *p* < .001, ${\eta_{p}}^{2}$ = .116, and block, *F*(3,398) = 49.48, *p* < .001, ${\eta_{p}}^{2}$ = .272, were qualified by CS × block, *F*(3, 398) = 25.54, *p* < .001, ${\eta_{p}}^{2}$ = .161, and CS‐type × CS × block interactions, *F*(3, 398) = 3.63, *p* = .013, ${\eta_{p}}^{2}$ = .027. Follow‐up analyses of the three‐way interaction indicate that in both groups responses after CS+ were larger than after CS− on blocks 1 and 2, both *F*(1,400) > 4.82, *p* < .030, ${\eta_{p}}^{2}$ > .011, but not on blocks 3 and 4, both *F*(1,400) < 2.37, *p* > .124, ${\eta_{p}}^{2}$ < .007, and there was no difference in differential responding across blocks between the groups. The change in responding after the CSs differed across blocks between the groups with responding after CS+ increasing from block 3 to 4 in participants trained with faces, but not in participants trained with shapes. All other effects were non‐significant, *F* < 2.79, *p* > .095, ${\eta_{p}}^{2}$ < .008.

### 3.5 Rated CS pleasantness

**FIGURE 4:**
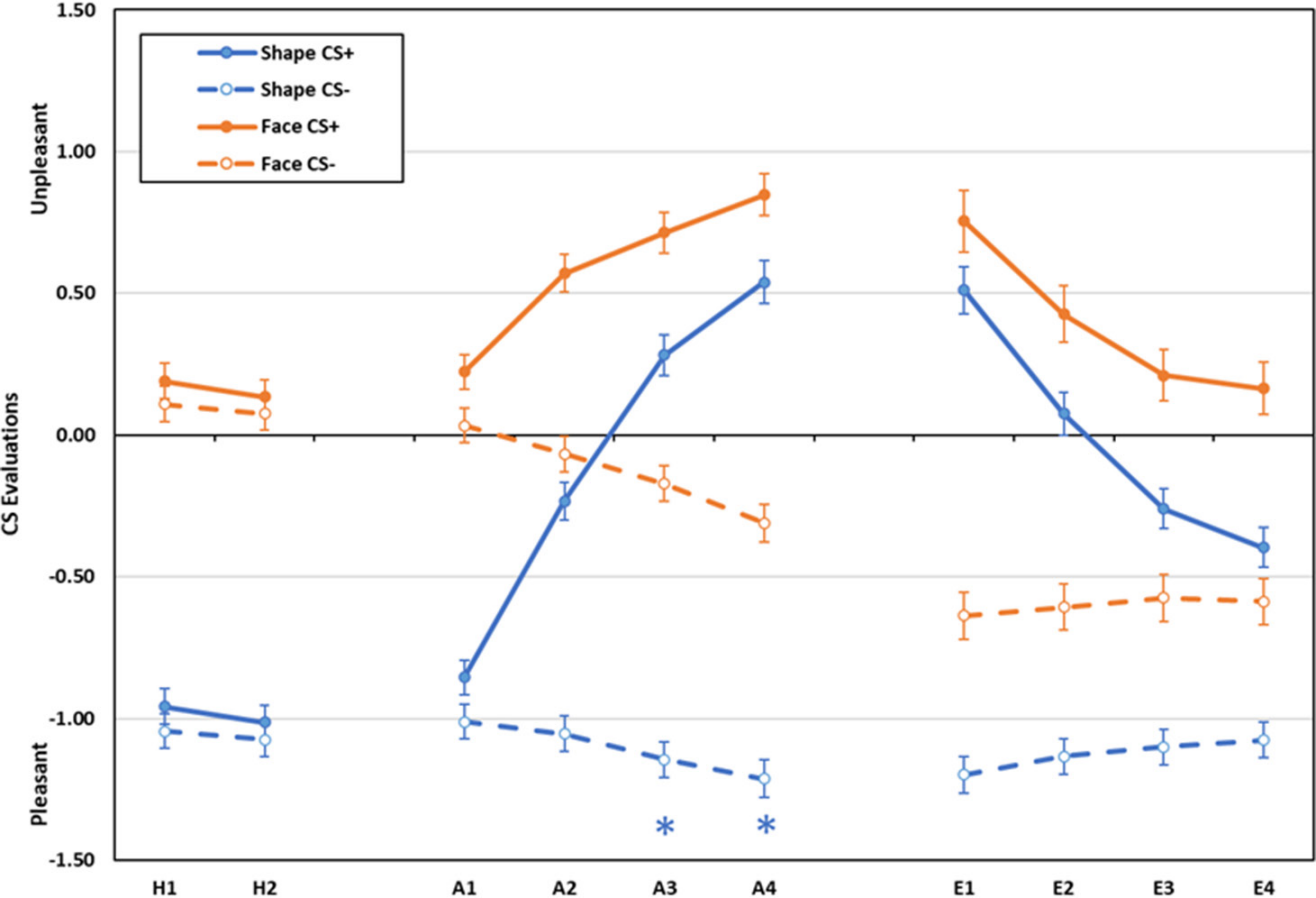
Rated CS pleasantness during habituation, acquisition, and extinction training as a function of CS‐type (shapes vs. neutral faces), CS (CS+ vs. CS−), and blocks of 2 trials (error bars represent standard errors of the mean; 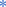 indicate larger differential responding in group shapes)

Scores for rated CS pleasantness during habituation, acquisition and extinction are displayed in Figure 4. During habituation, shapes were rated as more pleasant than faces, main effect CS‐type, *F*(1,589) = 240.94, *p* < .001, ${\eta_{p}}^{2}$ = .290, and pleasantness ratings increased across blocks, main effect for block, *F*(1,589) = 9.79, *p* = .002, ${\eta_{p}}^{2}$ = .016. All other effects were non‐significant, *F* < 3.08, *p* > .079, ${\eta_{p}}^{2}$ < .006.

During acquisition, main effects for CS‐type, *F*(1,589) = 150.13, *p* < .001, ${\eta_{p}}^{2}$ = .203, CS, *F*(1,589) = 274.01, *p* < .001, ${\eta_{p}}^{2}$ = .318, and block, *F*(3,587) = 65.90, *p* < .001, ${\eta_{p}}^{2}$ = .252, were qualified by CS‐type × CS, *F*(1,589) = 9.17, *p* = .003, ${\eta_{p}}^{2}$ = .015, CS‐type × block, *F*(3,587) = 23.03, *p* < .001, ${\eta_{p}}^{2}$ = .105, CS × block, *F*(3,587) = 132.06, *p* < .001, ${\eta_{p}}^{2}$ = .403 and CS‐type × CS × block interactions, *F*(3,587) = 10.42, *p* < .001, ${\eta_{p}}^{2}$ = .051. Evaluation of the CS− was more positive than of CS+ in both groups on all blocks, all *F*(1,589) > 6.20, *p* < .014, ${\eta_{p}}^{2}$ > .090, but differential evaluations of CS+ and CS− were larger for shapes than for faces on blocks 3 and 4, both *F*(1,589) > 16.61, *p* < .001, ${\eta_{p}}^{2}$ > .026, but not on blocks 1 and 2, both *F*(1,589) < 2.67, *p* > .102, ${\eta_{p}}^{2}$ < .006.

During extinction, main effects of CS‐type, *F*(1,394) = 32.72, *p* < .001, ${\eta_{p}}^{2}$ = .077, CS, *F*(1,394) = 237.12, *p* < .001, ${\eta_{p}}^{2}$ = .376, and block, *F*(3,392) = 37.07, *p* < .001, ${\eta_{p}}^{2}$ = .221, were qualified by CS × block, *F*(3, 392) = 56.62, *p* < .001, ${\eta_{p}}^{2}$ = .302, and CS‐type × CS × block interactions, *F*(3, 392) = 3.44, *p* = .017, ${\eta_{p}}^{2}$ = .026. Again, evaluation of the CS− was more pleasant on all blocks in both groups, all *F*(1,394) > 53.67, *p* < .001, ${\eta_{p}}^{2}$ > .119, but differential evaluations did not differ between CS‐types, all *F*(1,394) < 3.33, *p* > .068, ${\eta_{p}}^{2}$ < .009. The three‐way interaction reflects that across blocks, evaluations of the CS+ became more positive in group Shapes (block 1 < block 2 < block 3 < block 4, all *p* < .001), but not in group Faces (block 1 < block 2 < block 3 = block 4, all *p* < .001). Evaluations of CS− were more positive on block 1 than on block 4 in group Shapes (*p* = .003) but did not change in group faces (all *p* > .202). All other effects were non‐significant, *F* < 1.32, *p* > .269, ${\eta_{p}}^{2}$ < .011.

## 4 DISCUSSION

The current analysis yielded clear evidence for differential fear conditioning as indexed by electrodermal responses and ratings of CS pleasantness in participants trained with neutral shape and face conditional stimuli. Differences in fear conditioning as a function of the nature of the conditional stimuli used were evident but were limited to second interval electrodermal responses during acquisition and ratings of CS pleasantness during acquisition and extinction. There was no evidence for differences in fear conditioning between the two rather large samples in the most frequently reported index of human fear conditioning, electrodermal first interval responses.

In the second interval electrodermal responses, which are said to index anticipatory processes more than first interval responses which have been argued to reflect the processing of CS novelty or significance (Luck & Lipp, 2016; Öhman, 1983) differential responding was larger in the face than shape group in block 1 of acquisition, whereas the inverse held for blocks 2 and 4 of acquisition. The difference early during acquisition could be due to differences in stimulus discriminability between groups with discrimination between CS+ and CS− easier for neutral faces than for neutral shapes. This interpretation is consistent with the finding of a higher percentage of participants in the shape group failing to report the experimental contingencies in a delayed post‐experimental questionnaire. Alternatively, it may be easier to attribute agency to pictures of people than to shapes and this could explain the negative outcomes associated with the stimuli. This advantage seems short‐lived, however, with shapes eliciting larger differential second interval electrodermal responses later during acquisition. The difference between groups seen on the last block of extinction, whereby participants trained with faces showed differential responding again, is difficult to interpret and may be indicative of participants expecting a reversal of the contingencies after repeated presentations of the CSs alone, a treatment that may be seen to resemble the habituation training that preceded acquisition.

The current findings reinforce the notion that electrodermal responses measured in different latency windows to some extent reflect different underlying psychological processes (Luck & Lipp, 2016; Prokasy & Kumpfer, 1973). It should be noted, however, that they are not pure measures, as enhanced unconditional stimulus anticipation, for instance, may enhance the signal value of a CS and thus electrodermal first interval responses. Conversely, the nature of the CS may enhance the perceived belongingness between CS and unconditional stimulus and hence the extent of unconditional stimulus anticipation and second interval responses. Nevertheless, the emergence of differences in electrodermal second interval responses in the presence of a virtually identical pattern in first interval responses should be taken as an encouragement to quantify more than just one electrodermal response component.

Resembling the results for second interval electrodermal responses, differential ratings of CS pleasantness late during acquisition were larger for shapes than for faces. This difference seems largely driven by differences between the groups in the trajectory of the CS+ ratings across acquisition whereas changes in the ratings of CS− across acquisition were similar across groups. The fact that the pleasantness of the shape CS+ changed more than the pleasantness of the face CS+ may be due to the baseline difference in rated pleasantness between stimuli observed during habituation. Here, shapes were rated as mildly pleasant whereas neutral faces were rated as more unpleasant. This may have provided a larger range for change in rated pleasantness given the limits of the current experimental setting. The notion that it may be easier to shift the valence of rather arbitrary stimuli such as shapes than of more meaningful ones such as faces is also consistent with the results from extinction. Here, the shape CS− lost some of the positive valence it had acquired during acquisition, whereas the evaluations of the face CS− did not change. Relatedly, and perhaps due to the larger change seen during acquisition, evaluations of the shape CS+ continued to change in the later blocks of extinction which was not the case for the face CS+. It should be noted, however, that these differences between CS groups were subtle and may not have been observed in an analysis that was less well powered than the current one.

## 5 EXPERIMENT 2

### 5.1 Method

#### 5.1.1 Data and participants

Data from two studies (Lucas et al., 2018; one currently unpublished [HREC approval number HRE2019‐0044]) using either Neutral or angry faces as CSs were compiled for Experiment 2. One hundred and fifty‐seven participants (48 trained with angry faces; 50 males and 107 females, aged 17–62; mean = 22.45, *SD* = 7.58), provided electrodermal and evaluation data from acquisition and 61 participants (24 trained with angry faces; 26 males and 35 females, aged 18–55; mean = 22.46, *SD* = 6.77) provided electrodermal and evaluation data from extinction. Habituation data from one participant trained with angry faces were not available. Participants were sourced from university cohorts, participated in exchange for course credits or a cash reimbursement, and provided informed consent.

#### 5.1.2 Apparatus and materials

The neutral face CSs were four pictures of adult male Caucasians with neutral expressions taken from the NimStim database (poses CA_C of models 24, 28, 34, and 36; Tottenham et al., 2009) presented on a black background. The angry face CSs were four pictures of adult male Caucasians with angry expressions from the same database (poses AN_O of models 20, 23, 32, and 34) presented on a gray background. Conditional stimuli in Experiment 2 were presented for 8 s. All other apparatus and materials were the same as for Experiment 1, including the use of a 200 ms electro‐tactile stimulus that was pulsed at 50 Hz through a Grass SD9 stimulator or a sequence of three 2 ms electro‐tactile stimuli generated by a Digitimer DS7A stimulator unit presented 16 ms apart (perceived as one discrete stimulus) as the experimental US.

#### 5.1.3 Procedure

Procedures up until the beginning of acquisition were identical to Experiment 1. During acquisition, 12 CS+s and 12 CS−s were presented, with the CS+ followed by the co‐terminating electric shock on 50% (rather than 100%) of the trials. In Experiment 2, there were 16 trials each for CS+ and CS− during extinction. All other procedures were identical to Experiment 1.

Three of the five experimental groups from Experiment 2 had manipulations during extinction, hence only data from participants who received standard extinction training were included in the analyses.

#### 5.1.4 Data scoring and analysis

Scoring of skin conductance responses and CS pleasantness employed the same rationale as for Experiment 1, however, latency windows were modified to account for the longer CS durations. First interval responses (FIR) were scored as those starting within 1–4 s following CS onset, second interval responses (SIR) were scored as those starting within 4–9 s following CS onset, and third interval responses (TIR) were scored as those starting 9–13 s following CS onset (Luck & Lipp, 2016; Prokasy & Ebel, 1967). All other details were the same as for Experiment 1 with the block factors having more levels (6 and 8 for acquisition and extinction, respectively). Third interval responses during acquisition were analyzed for trials with and without unconditional stimuli separately using a block factor with 3 levels.

## 6 RESULTS

### 6.1 Preliminary analyses

The two groups of participants did not differ in terms of gender distribution (neutral group: 78 female; 31 male; angry group: 29 female; 19 male), *χ*2 = 1.91, *p* = .167, rated US pleasantness (neutral group: *M* = −1.55, *SD* = 0.65; angry group: *M* = −1.67, *SD* = 1.04), *t*(155) = 0.88, *p* = .379, or number of participants who were aware of the CS–US contingency (neutral group: 95 of 109; angry group: 37 of 48), *χ* ^2^ = 2.53, *p* = .112. However, the group trained with angry face CSs had a higher number of skin conductance responses during baseline (neutral group: *M* = 18.18, *SD* = 12.54; angry group: *M* = 32.63, *SD* = 18.09), *t*(155) = 5.77, *p* < .001, were older (neutral group: *M* = 21.06, *SD* = 5.33; angry group: *M* = 25.60, *SD* = 10.53), *t*(155) = 3.59, *p* < .001, and had smaller maximal skin conductance responses (neutral group: *M* = 2.15 μS, *SD* = 0.52; angry group: *M* = 1.67 μS, *SD* = 0.56), *t*(155) = 5.15, *p* < .001. We assessed whether these group differences affected the outcomes of the current study by running preliminary analyses that included these variables as covariates and report if the inclusion of a covariate affected effects involving the between groups factor.

### 6.2 Electrodermal first interval responses

**FIGURE 5:**
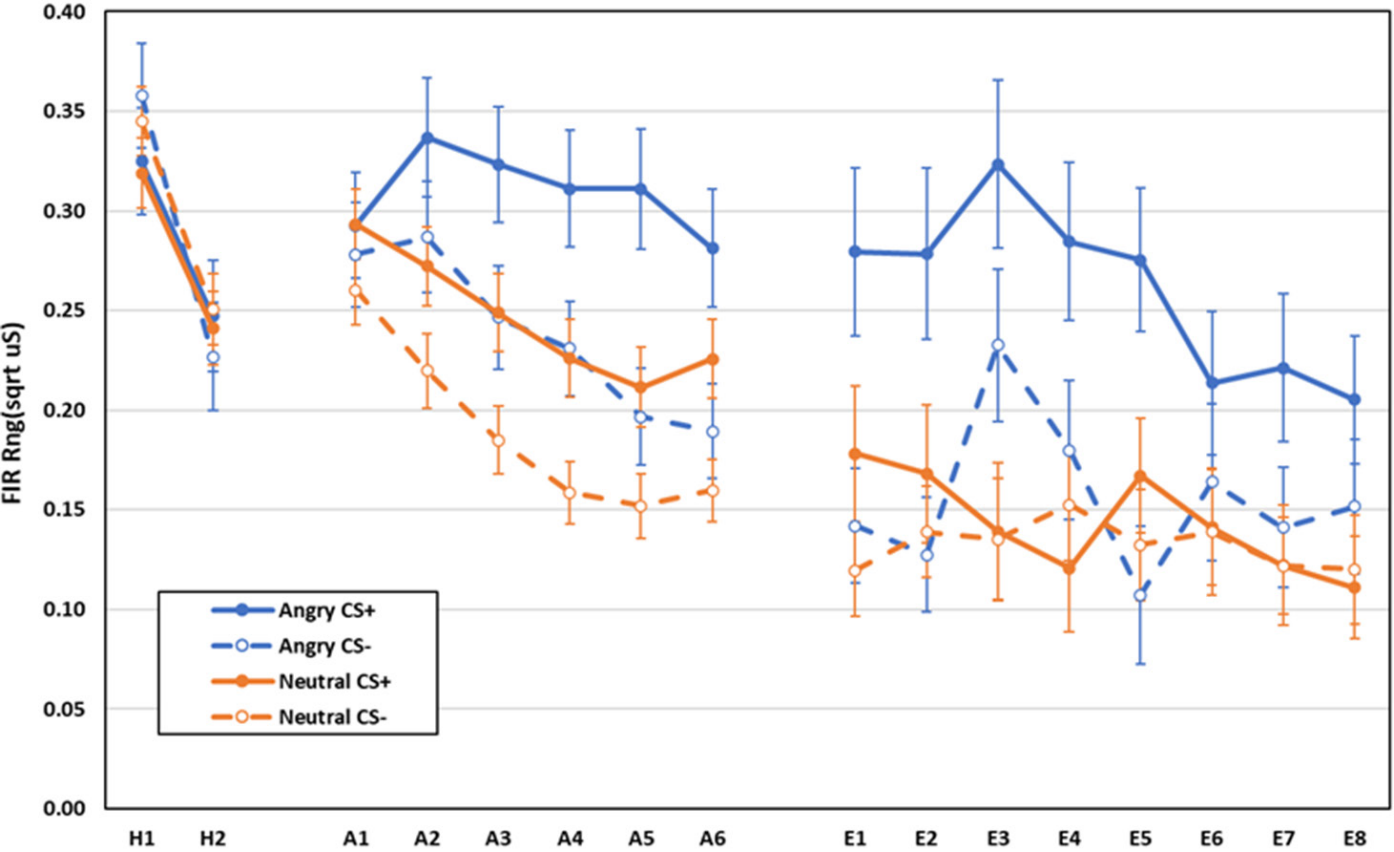
Electrodermal first interval responses during habituation, acquisition, and extinction training as a function of CS‐type (angry vs. neutral faces), CS (CS+ vs. CS−), and blocks of 2 trials (error bars represent standard errors of the mean)

Electrodermal first interval responses during habituation, acquisition, and extinction are displayed in Figure 5. Electrodermal first interval responses declined between blocks, *F*(1,154) = 57.33, *p* < .001, ${\eta_{p}}^{2}$ = .271, but no other effects were significant, all *F* < 2.22, *p* > .138, ${\eta_{p}}^{2}$ < .015.

During acquisition, responses were larger to angry than to neutral faces, main effect CS‐type, *F*(1,155) = 6.04, *p* = .015, ${\eta_{p}}^{2}$ = .038,^3^ and to CS+, main effect CS, *F*(1,155) = 45.21, *p* < .001, ${\eta_{p}}^{2}$ = .226, and declined across blocks, main effect of block, *F*(5,151) = 5.84, *p* < .001, ${\eta_{p}}^{2}$ = .162. All other effects were not significant, *F* < 2.14, *p* > .062, ${\eta_{p}}^{2}$ < .067 (largest effect: CS × block interaction).

During extinction, main effects for CS‐type, *F*(1,59) = 5.57, *p* = .022, ${\eta_{p}}^{2}$ = .086,^4^ and CS, *F*(1,59) = 11.81, *p* = .001, ${\eta_{p}}^{2}$ = .167, were qualified by a CS‐type × CS interaction, *F*(1,59) = 7.71, *p* = .007, ${\eta_{p}}^{2}$ = .116. Follow‐up analyses revealed that the CS+ elicited larger responses than the CS− in the angry face group, *F*(1,59) = 15.92, *p* < .001, ${\eta_{p}}^{2}$ = .212, but not in the neutral face group, *F*(1,59) = 0.276, *p* = .601, ${\eta_{p}}^{2}$ = .005. All other effects were non‐significant, *F* < 1.77, *p* > .112, ${\eta_{p}}^{2}$ < .190.

### 6.3 Electrodermal second interval responses

**FIGURE 6:**
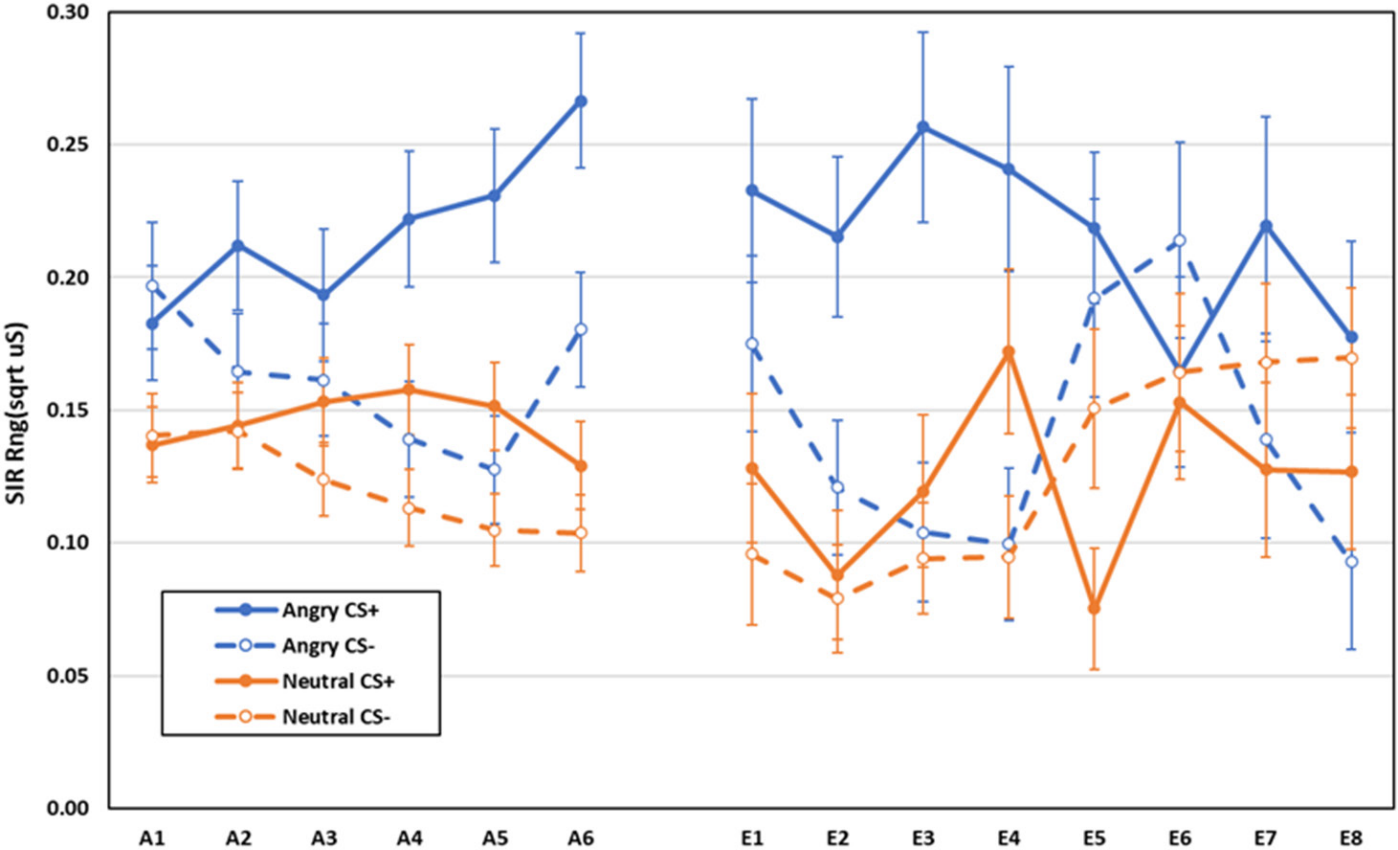
Electrodermal second interval responses during acquisition and extinction training as a function of CS‐type (angry vs. neutral faces), CS (CS+ vs. CS−), and blocks of 2 trials (error bars represent standard errors of the mean)

Electrodermal second interval responses during acquisition and extinction are displayed in Figure 6. During acquisition, a main effect for CS, *F*(1,59) = 21.67, *p* < .001, ${\eta_{p}}^{2}$ = .123, was qualified by a CS × block interaction, *F*(5,151) = 3.47, *p* < .001, ${\eta_{p}}^{2}$ = .103. Breakdown of the interaction revealed no difference between CS+ and CS− on blocks 1–3, all *F*(1,155) < 3.13, *p* > .078, ${\eta_{p}}^{2}$ < .021, but larger responses to CS+ than to CS− on Blocks 4–6, all *F*(1,155) > 10.74, *p* < .002, ${\eta_{p}}^{2}$ > .064. All other effects were non‐significant, all *F* < 3.50, *p* > .063, ${\eta_{p}}^{2}$ < .023 (largest effect: CS‐type × CS interaction).

During extinction, the analysis yielded main effects of CS‐type, *F*(1,59) = 5.75, *p* = .020, ${\eta_{p}}^{2}$ = .089 (Footnote 4), and CS, *F*(1,59) = 7.10, *p* = .010, ${\eta_{p}}^{2}$ = .107, which were qualified by CS‐type × CS, *F*(1,59) = 8.48, *p* = .005, ${\eta_{p}}^{2}$ = .126, and CS × block interactions, *F*(7, 53) = 3.26, *p* = .006, ${\eta_{p}}^{2}$ = .301. All other effects were non‐significant, *F* < 1.64, *p* > .146, ${\eta_{p}}^{2}$ < .178. Follow‐up analyses of the CS‐type × CS interaction revealed differential responding for angry faces, *F*(1,59) = 12.82, *p* = .001, ${\eta_{p}}^{2}$ = .178, but not for neutral faces: *F*(1,59) = 0.039, *p* = .845, ${\eta_{p}}^{2}$ = .001. Breakdown of the CS × block revealed that CS+ elicited larger responses than CS− on blocks 2–4, all *F*(1,59) > 4.69, *p* < .035, ${\eta_{p}}^{2}$ > .073, but not on blocks 1 and 5–8, all *F*(1,59) < 2.01, *p* > .161, ${\eta_{p}}^{2}$ < .034.

### 6.4 Electrodermal third interval responses

**FIGURE 7:**
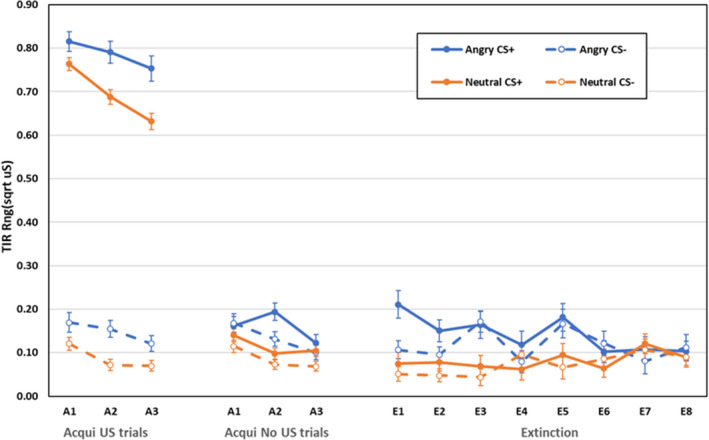
Electrodermal third interval responses during acquisition (trials with and without USs) and extinction training as a function of CS‐type (angry vs. neutral faces), CS (CS+ vs. CS−), and blocks of 2 trials (error bars represent standard errors of the mean)

Electrodermal first interval responses during acquisition and extinction are displayed in Figure 7.

### 6.5 Acquisition trials with shock

Responses were larger in the angry face group, main effect CS‐type, *F*(1,155) = 20.75, *p* < .001, ${\eta_{p}}^{2}$ = .118, larger after CS+, main effect CS, *F*(1,155) = 1999.15, *p* < .001, ${\eta_{p}}^{2}$ = .928, and declined across blocks, main effect of block, *F*(5,155) = 20.69, *p* < .001, ${\eta_{p}}^{2}$ = .212. All other effects were not significant: *F* < 2.23, *p* > .111, ${\eta_{p}}^{2}$ < .029.

### 6.6 Acquisition trials without shock

As shown in the middle panel of Figure 7, during acquisition, third interval responses were larger after CS+ than after CS−, main effect CS, *F*(1,155) = 8.68, *p* = .004, ${\eta_{p}}^{2}$ = .053. Main effects for CS‐type, *F*(1,155) = 10.0, *p* = .002, ${\eta_{p}}^{2}$ = .061 (Footnote 3), and block, *F*(5,155) = 8.08, *p* < .001, ${\eta_{p}}^{2}$ = .095, were qualified by a CS‐type × block interaction, *F*(5,155) = 3.82, *p* = .024, ${\eta_{p}}^{2}$ = .047.^5^ Responses after angry faces were larger than after neutral faces on block 2, *F*(1,155) = 18.23 *p* < .001, ${\eta_{p}}^{2}$ = .105, but not on blocks 1 or 3, both *F*(1,155) < 2.79, *p* > .096, ${\eta_{p}}^{2}$ < .019. All other effects were non‐significant, *F* < 2.23, *p* > .111, ${\eta_{p}}^{2}$ < .029.

### 6.7 Extinction

During extinction, a main effect of CS‐type, *F*(1,59) = 8.05, *p* = .006, ${\eta_{p}}^{2}$ = .120 (Footnote 3), was qualified by a CS‐type × block interaction, *F*(7, 53) = 3.53, *p* = .003, ${\eta_{p}}^{2}$ = .318. Responses after angry faces were larger compared to neutral faces during blocks 1–3 and 5, all *F*(1,59) > 7.49, *p* < .008, ${\eta_{p}}^{2}$ > .112, but there was no difference on blocks 4, 6–8, all *F*(1,59) < 1.85, *p* > .178, ${\eta_{p}}^{2}$ < .031. All other effects were non‐significant, *F* < 2.75, *p* > .102, ${\eta_{p}}^{2}$ < .163.

### 6.8 Rated CS pleasantness

**FIGURE 8:**
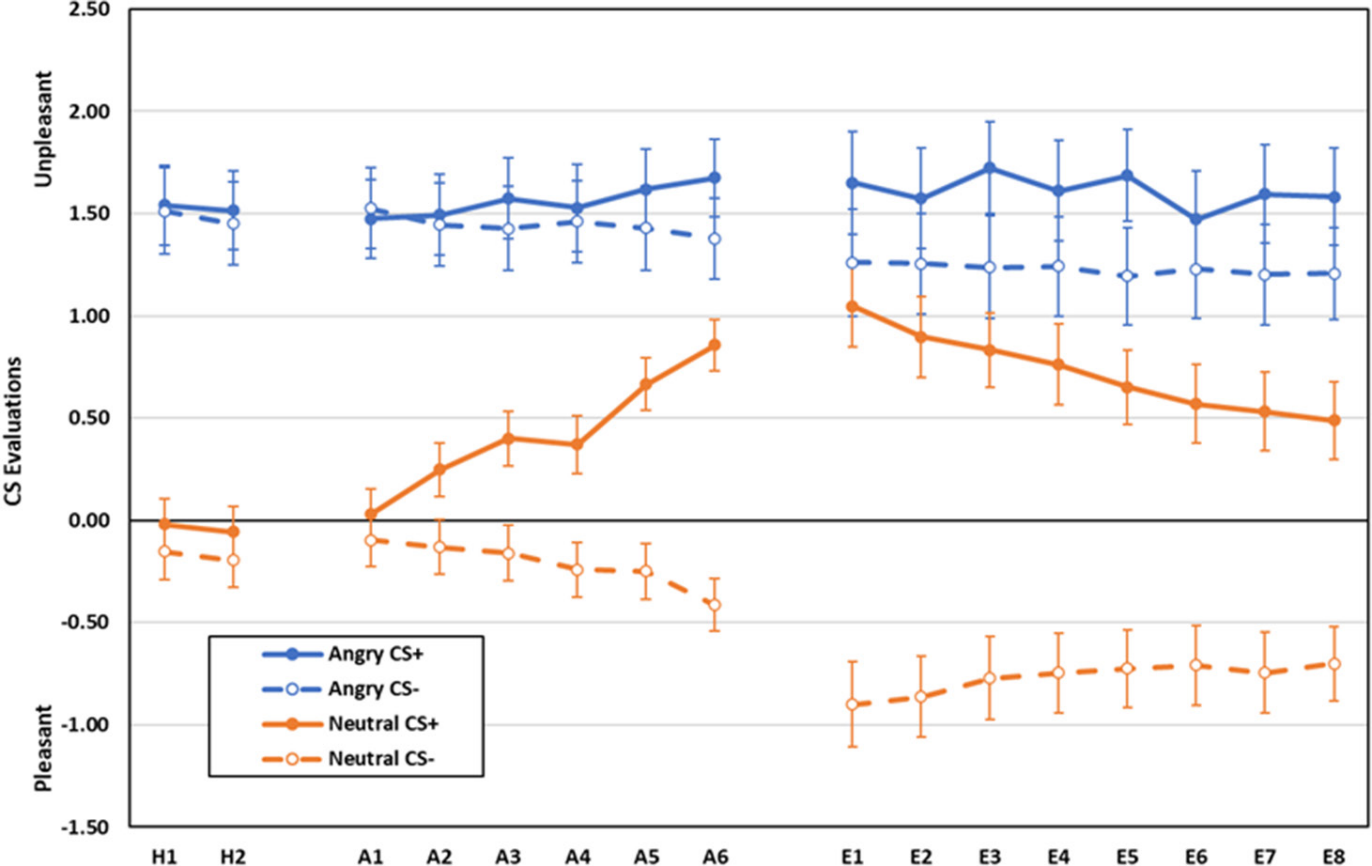
Rated CS pleasantness during habituation, acquisition, and extinction training as a function of CS‐type (angry vs. neutral faces), CS (CS+ vs. CS−), and blocks of 2 trials (error bars represent standard errors of the mean)

Scores for rated CS pleasantness during habituation, acquisition and extinction are displayed in Figure 8. During habituation, neutral faces were rated as more pleasant than angry faces, main effect for CS‐type, *F*(1,154) = 67.32, *p* < .001, ${\eta_{p}}^{2}$ = .304. All other effects were non‐significant, *F* < 1.60, *p* > .207, ${\eta_{p}}^{2}$ < .011.

During acquisition, main effects for CS‐type, *F*(1,155) =62.15, *p* < .001, ${\eta_{p}}^{2}$ = .286, and CS, *F*(1,155) = 7.75, *p* = .006, ${\eta_{p}}^{2}$ = .048, were qualified by CS × block, *F*(5,151) = 7.55, *p* < .001, ${\eta_{p}}^{2}$ = .200, and CS‐type × CS × block, *F*(3,587) = 2.35, *p* = .044, ${\eta_{p}}^{2}$ = .072,^6^ interactions. Breakdown of the CS‐type × CS × block revealed that there was no differential evaluation of CS+ and CS− for angry faces, all *F*(1,155) < 1.25, *p* > .266, ${\eta_{p}}^{2}$ < .009, whereas neutral face CSs+  were evaluated as more negative than CSs‐ on blocks 2–6, all *F*(1,155) > 6.05, *p* < .016, ${\eta_{p}}^{2}$ > .037, but not on block 1, *F*(1,155) =0.77, *p* = .381, ${\eta_{p}}^{2}$ = .005.

During extinction, main effects of CS‐type, *F*(1,59) = 34.43, *p* < .001, ${\eta_{p}}^{2}$ = .369, CS, *F*(1,59) = 30.46, *p* < .001, ${\eta_{p}}^{2}$ = .341, and block, *F*(7,53) = 2.42, *p* = .032, ${\eta_{p}}^{2}$ = .242, were qualified by CS‐type × CS, *F*(1, 59) = 10.65, *p* = .002, ${\eta_{p}}^{2}$ = .153, and CS × block interactions, *F*(3, 392) = 56.62, *p* < .001, ${\eta_{p}}^{2}$ = .302. The CS‐type × CS interaction was due to higher pleasantness ratings for CS‐ than CS+ for neutral, *F*(1,59) = 49.02, *p* < .001, ${\eta_{p}}^{2}$ = .454, but not angry faces, *F*(1,59) = 2.10, *p* = .153, ${\eta_{p}}^{2}$ = .034. The CS × block interaction reflects smaller differential evaluations on block 8 than on block 1 (*p* = .005). All other effects were non‐significant, all *F* < 1.99, *p* > .074, ${\eta_{p}}^{2}$ < .208.

## 7 DISCUSSION

Experiment 2 replicated past findings that the extinction of electrodermal fear responses conditioned to angry faces is slower than the extinction of electrodermal fear responses conditioned to neutral faces (Dimberg & Öhman, 1983, 1996; Esteves et al., 1994; Hamm et al., 1989; Mazurski et al., 1996; Öhman & Dimberg, 1978; Rowles et al., 2012). It extends on this work in that the difference in extinction was shown after conditioning in a partially reinforced design, not the 100% reinforcement schedules most frequently used in research on prepared learning. The use of a partial reinforcement schedule may also explain the size of the observed difference which is considerably bigger than those reported in the past. Contrary to expectation, there was no difference between the groups in the speed at which differential conditioning was acquired. Such a difference had been observed in a completely within‐participant design employing a partially reinforced conditioning procedure by Ho and Lipp (2014) for fear conditioned to pictures of fear‐relevant and non‐fear‐relevant animals. It should be noted that Ho and Lipp (2014) found a significant between‐group difference only during the first two blocks of acquisition training. A similar analysis of the current data did not yield a significant result. It may be that between stimulus differences are best observed in completely within‐subject designs, a suggestion supported by the observation that Ho and Lipp (2014) observed larger electrodermal responses to fear‐relevant than non‐fear‐relevant animals during habituation whereas there was no difference between the CS‐types observed during habituation in the current study.

Relatedly, we previously found larger skin conductance responses to fear‐relevant anger compared to non‐fear‐relevant happy face stimuli during habituation when habituation was preceded by a shock work‐up procedure (Lipp et al., 2015). This result was not replicated in the current study, with angry and neutral face CSs showing equivalent responses during habituation, despite habituation following a shock work‐up. The critical difference between these studies again appears to be the use of a within‐subjects design, which was not used in the current study. Future research should carefully evaluate the comparability of findings from within‐subjects fear conditioning experiments to between‐subjects designs, as our present data seem to suggest that they may not be directly comparable in all situations (see also Luck et al., 2020).

Ratings of CS pleasantness failed to provide evidence for differential conditioning in participants trained with angry faces. This pattern of results emerged in the post experimental questionnaire as well in which participants were asked to provide ratings of CS pleasantness on a seven‐point Likert scale. Here, the CS‐type (neutral vs angry faces) × CS (CS+, CS−) mixed factorial ANOVA yielded main effects for CS‐type, *F*(1,59) = 29.14, *p* = .001, ${\eta_{p}}^{2}$ = .172, and CS, *F*(1,59) = 24.34, *p* < .001, ${\eta_{p}}^{2}$ = .292, and a CS‐type × CS interaction, *F*(1,59) = 12.25, *p* = .001, ${\eta_{p}}^{2}$ = .172. The interaction reflects differential CS evaluations in group Neutral, *F*(1,59) = 45.19, *p* < .001, ${\eta_{p}}^{2}$ = .434, but not in group Angry, *F*(1,59) = 0.85, *p* = .361, ${\eta_{p}}^{2}$ = .014. Thus, the failure to rate the CS− as more pleasant than the CS+ was not limited to the online assessments taken during throughout the experiment. While unexpected, this finding seems due to the low baseline pleasantness of the angry face stimuli which was not subject to change for a CS− that predicted the absence of the US.

## 8 GENERAL DISCUSSION

Methodological decisions in fear conditioning research should be carefully guided by empirical evidence. However, such evidence is frequently not available. In a re‐analysis of data from nine studies from our laboratories, we tested whether skin conductance and CS pleasantness evaluations during fear acquisition and extinction differed when the CSs were shapes compared to neutral faces (Experiment 1), and when CSs were neutral compared to angry faces (Experiment 2). We hypothesized that neutral face and shape CSs would show equivalent acquisition and extinction and that differential conditioning to angry faces would be acquired faster and be resistant to extinction compared to conditioning with neutral faces.

In contrast to our hypotheses, there was some evidence for stronger differential fear acquisition for neutral shape than face CSs in electrodermal second interval responses and more clearly ratings of CS pleasantness, but not in the most commonly used measure, first interval electrodermal responses. One potential explanation for these results may be that pairing shapes or faces with an aversive event generated different prediction errors. Theories of fear learning stipulate that both acquisition and extinction learning are strongest when the greatest violation of one's expectations is experienced (Li & McNally, 2014; Rescorla & Wagner, 1972). Shapes were perceived as more pleasant than neutral faces during habituation and experiencing an aversive stimulus after the relatively more pleasant CS materials may have resulted in a larger prediction error than experiencing the same aversive stimulus after a less pleasant CS. Given the larger extent of differential CS pleasantness acquired by shape CSs during acquisition, extinction was also more pronounced for shapes compared to faces with changes in rated CS pleasantness evident for CS+ and CS−. These findings suggest that shape CSs may support the stronger acquisition and extinction effects, though the differences in effect sizes between the groups were small and most differences in second interval response conditioning would not survive Bonferroni adjustments of the significance levels for follow‐up testing. Moreover, significant differences were not present in electrodermal first interval responses, the most frequently reported index of fear conditioning. This suggests that while detectable in a well‐powered study, differences in fear conditioning between shape and neutral face CSs are limited and that either stimulus material is suited for use in studies of human fear conditioning.

Marked differences emerged for fear conditioned to angry versus neutral faces. Replicating past findings (for a review see Mallan et al., 2013), fear conditioning as indexed by electrodermal first, as well as second interval responses, was slower to extinguish when trained with angry than with neutral faces. Moreover, differential evaluations of CS+ and CS− pleasantness were not observed for the angry face stimuli. This may suggest that it is difficult to change the baseline evaluations of some stimuli when they are subjected to aversive fear conditioning contingencies. On the other hand, it should be noted that angry faces, while unpleasant, are not overly so if seen, for instance in the context of a broader range of emotional stimuli like that provided by the International Affective Picture System (Lang et al., 2008). Here, angry faces are rated as moderately aversive with a medium level of arousal. More work seems required to clarify the differential results observed across different measures of fear learning. However, based on the current findings, it seems advisable to employ angry face CSs only if they are the subject of investigation.

Several limitations of the current study should be noted. Although Experiment 1 was very well powered, the sample size in Experiment 2 was smaller, in particular for the assessment of extinction (i.e., 37 and 24 participants respectively). Moreover, although the experimental procedures in Experiments 1 and 2 were identical in terms of CSs and experimental design, data were collected at different time periods, by different researchers, and the experiments had some differences such as the nature and duration of the electrical stimulus, implying that there are some heterogeneities present in the compiled datasets. Finally, we did not employ independent tests of extinction learning such as assessments of the return of fear in different contexts or after a time delay. It is not impossible that such tests might have revealed further differences between the CS materials used. Despite these limitations, the data analyzed in this report should serve as a useful guide for researchers looking to design fear conditioning experiments in the future.

In conclusion, the results of Experiment 1 suggest that there is little difference between fear conditioned to shape and neutral face CSs, as indexed by the most frequently used index of human fear conditioning, electrodermal first interval responses. Differences did emerge for electrodermal second interval responses and rated CS pleasantness suggesting overall clearer results with shape CSs, i.e., stronger acquisition and a clear change in CS pleasantness ratings during extinction. Fear conditioned to angry face CSs seems more resistant to extinction than fear conditioned to neutral faces, as indexed by the primary measure of electrodermal first interval responses, as well as second interval responses, suggesting that angry faces should be used only as CSs in dedicated studies as their results may not generalize to other CS materials.

## AUTHOR CONTRIBUTIONS

**Luke J. Ney:** Investigation; writing – original draft; writing – review and editing. **Camilla C Luck:** Conceptualization; data curation; funding acquisition; investigation; writing – review and editing. **Allison Waters:** Conceptualization; funding acquisition; investigation; project administration; writing – review and editing.

## Footnotes

^1^
Inclusion of the covariates' age or maximum electrodermal response rendered the CS‐type × CS interaction nonsignificant. The remaining interactions involving the factor CS‐type remained significant.

^2^
Inclusion of the covariates' age, maximum electrodermal response, or contingency awareness rendered the main effect for CS‐type nonsignificant. Interactions involving the factor CS‐type remained significant.

^3^
The main effect was not significant if the number of spontaneous electrodermal responses during baseline was included as a covariate.

^4^
The main effect was not significant if the number of spontaneous electrodermal responses during baseline or the maximum electrodermal response displayed were included as covariates.

^5^
The interaction was not significant if the number of spontaneous electrodermal responses during baseline, the maximum electrodermal response, or participant age were included as covariates.

^6^
The interaction was not significant if the number of spontaneous electrodermal responses during baseline or participant age were included as covariates.
